# 2-Oxo-2*H*-chromen-4-yl 4-ethyl­benzoate

**DOI:** 10.1107/S2414314625007345

**Published:** 2025-08-19

**Authors:** Valentin Bationo, Abel Landry Tebily, Akoun Abou, Charles Bavouma Sombié, Rasmané Semdé, Abdoulaye Djandé

**Affiliations:** a Laboratory of Molecular Chemistry and Materials (LC2M), Research Team: Organic Chemistry and Phytochemistry, University Joseph KI-ZERBO, 03 BP 7021 Ouagadougou 03, Burkina Faso; b Joint Research and Innovation Unit for Engineering Sciences and Techniques (UMRI STI), Research Team: Instrumentation, Image and Spectroscopy, Félix Houphouet-Boigny National Polytechnic Institute, BP 1093 Yamoussoukro, Côte d’Ivoire; c Laboratory of Drug Development, Center of Training, Research and Expertise in Pharmaceutical Sciences (CFOREM), University Joseph KI-ZERBO, 03 BP 7021, Ouagadougou 03, Burkina Faso; University of Aberdeen, United Kingdom

**Keywords:** crystal structure, hydrogen bonding, π–π stacking, centrosymmetric dimer

## Abstract

In the title compound, C_18_H_14_O_4_, the dihedral angle between the coumarin moiety and the phenyl fragment is 63.46 (5)°. In the crystal, the mol­ecules are linked by weak C—H⋯O hydrogen bonds and aromatic π–π stacking inter­actions. A short C=O⋯π [O⋯π = 3.2667 (10) Å] contact is also observed.

## Abstract 2

In the title compound, the dihedral angle between the coumarin ring system and the phenyl ring is 63.46 (5)°. In the crystal, the mol­ecules are linked by weak C—H⋯O hydrogen bonds and aromatic π–π stacking inter­actions

## Graphical abstract

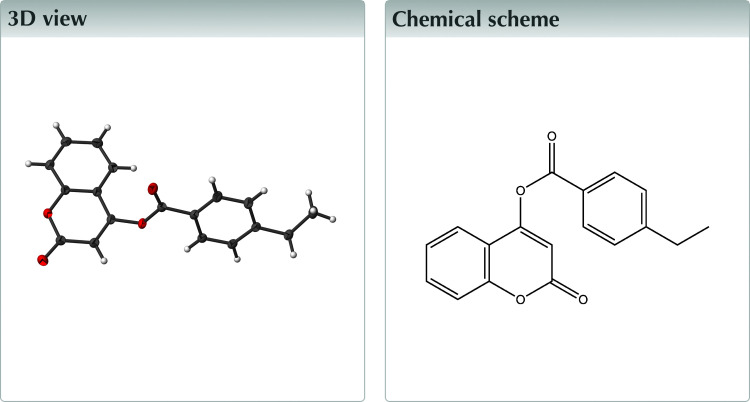

## Structure description

Coumarins are a broad class of over 800 naturally occurring chemicals and are frequently found in plants like sweet clover and tonka beans (Ziarani *et al.*, 2018 ▸). Some coumarins have therapeutic potential due to their wide range of biological activities (Akkol *et al.* 2020 ▸) such as anti-inflammatory action (Tuan Anh *et al.*, 2017 ▸; Tosun *et al.*, 2009 ▸). As part of our work in this area, we now describe the synthesis and structure of the title compound (**I**).

**Figure 1:**
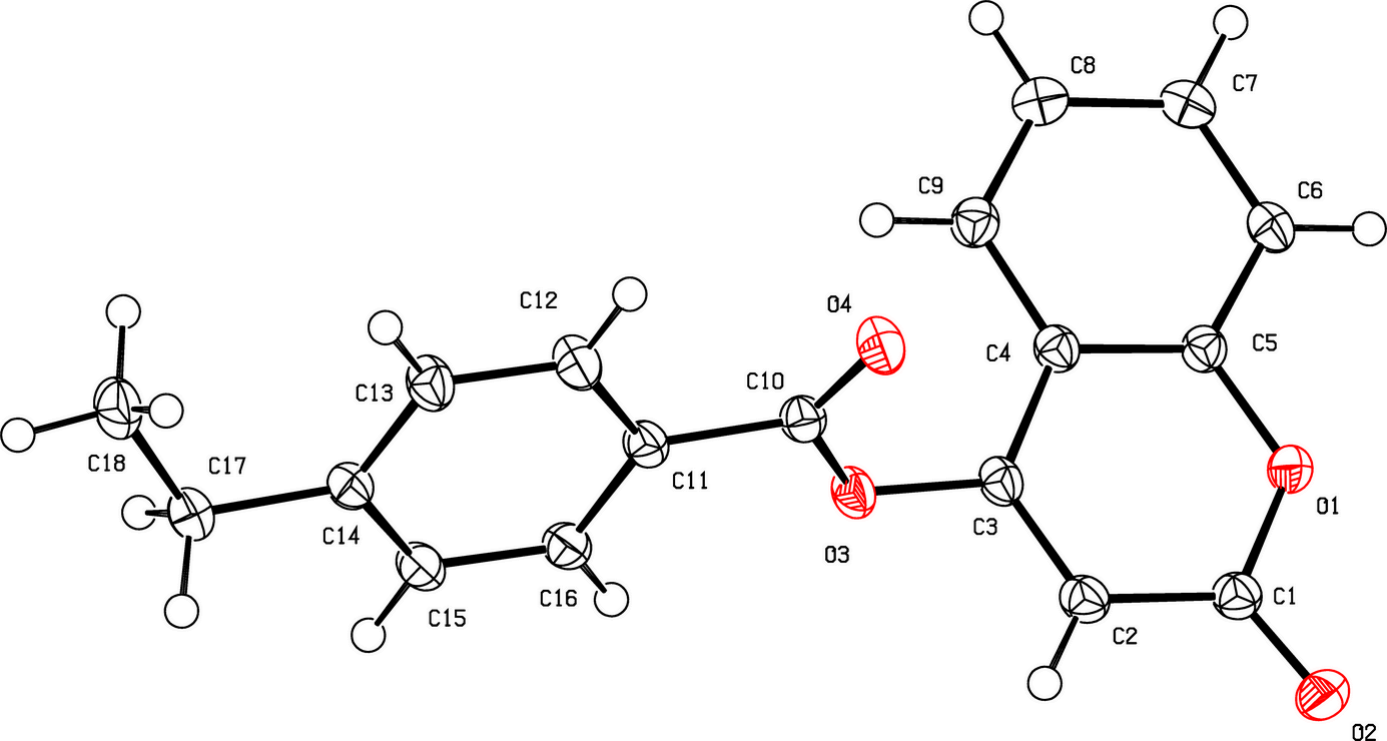
The mol­ecular structure of (**I**) with displacement ellipsoids drawn at the 50% probability level.

As expected, the C1–C9/O1 coumarin ring system in (**I**) (Fig. 1 ▸) is almost planar (r.m.s deviation = 0.004 Å) and is oriented at an angle of 63.46 (5)° with respect to the C11–C16 ring. Atom C18 lies close to the latter ring plane [deviation = −0.166 (1) Å]. The pyrone ring shows the usual asymmetric bond lengths for C3—C2 [1.3443 (15) Å] and C2—C1 [1.4508 (15) Å], which are shorter and longer, respectively, than would be expected for a C_ar_—C_ar_ bond (Gomes *et al.*, 2016 ▸; Koulabiga *et al.*, 2024 ▸).

**Figure 2:**
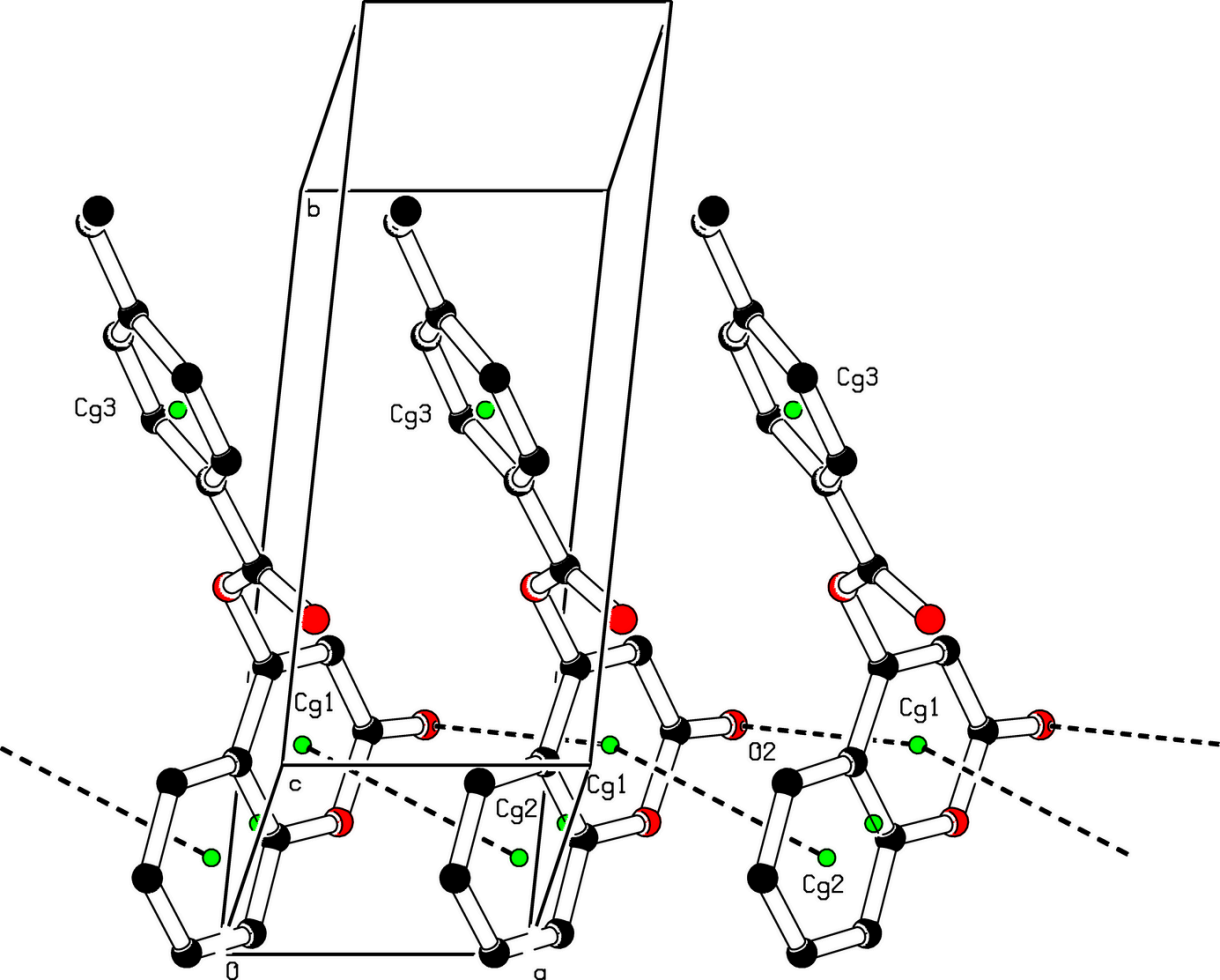
A view of the crystal packing of (**I**), showing C=O⋯π and π–π stacking inter­actions (dashed lines). The green dots are centroids of rings.

In the extended structure of (**I**) (Figs. 2 ▸ and 3 ▸), the mol­ecules are linked by weak C—H⋯O hydrogen bonds (Table 1 ▸). The C2—H2⋯O2 inter­action results in the formation of inversion dimers, which are characterized by an 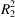
(8) graph-set motif. Subsequently, these dimers combine with the C9—H9⋯O4 and C16—H16⋯O2 hydrogen bonds to form an 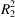
(16) graph-set motif. An aromatic π–π stacking inter­action is observed between the C1–C5/O1 and C4–C9 rings [centroid–centroid separation = 3.6514 (7) Å, slippage = 1.613 Å] and a short C=O⋯π contact of 3.2667 (10) Å occurs (Table 1 ▸).

## Synthesis and crystallization

To a solution of 4-ethyl­benzoyl chloride (0.95 ml, 6.2 mmol, 1 equiv.) in dried tetra­hydro­furan (30 ml) was added dried tri­ethyl­amine (2.6 ml, 3 equiv.) and 4-hy­droxy­coumarin (1.00 g, 6.17 mmol, 1 equiv.) in small portions over 30 min. The mixture was then refluxed for 4 h under stirring and poured into 40 ml of chloro­form. The solution was acidified with dilute hydro­chloric acid until its discoloration. The organic layer was extracted, concentrated in a vacuum until a slight cloudiness was obtained and then cooled in an ice bath. The resulting precipitate was filtered off with suction, washed with petroleum ether and recrystallized from a chloro­form–hexane solvent mixture (1:3) giving the title compound (1.12 g, yield 68%, m.p. 459–461 K). Colorless prisms appropriate for single-crystal X-ray diffraction analysis were obtained by slow evaporation of an acetone solution.

## Refinement

Crystal data, data collection and structure refinement details are summarized in Table 2 ▸.

## Supplementary Material

Crystal structure: contains datablock(s) I. DOI: 10.1107/S2414314625007345/hb4531sup1.cif

Structure factors: contains datablock(s) I. DOI: 10.1107/S2414314625007345/hb4531Isup3.hkl

Supporting information file. DOI: 10.1107/S2414314625007345/hb4531Isup3.cml

CCDC reference: 2481234

Additional supporting information:  crystallographic information; 3D view; checkCIF report

## Figures and Tables

**Figure 3:**
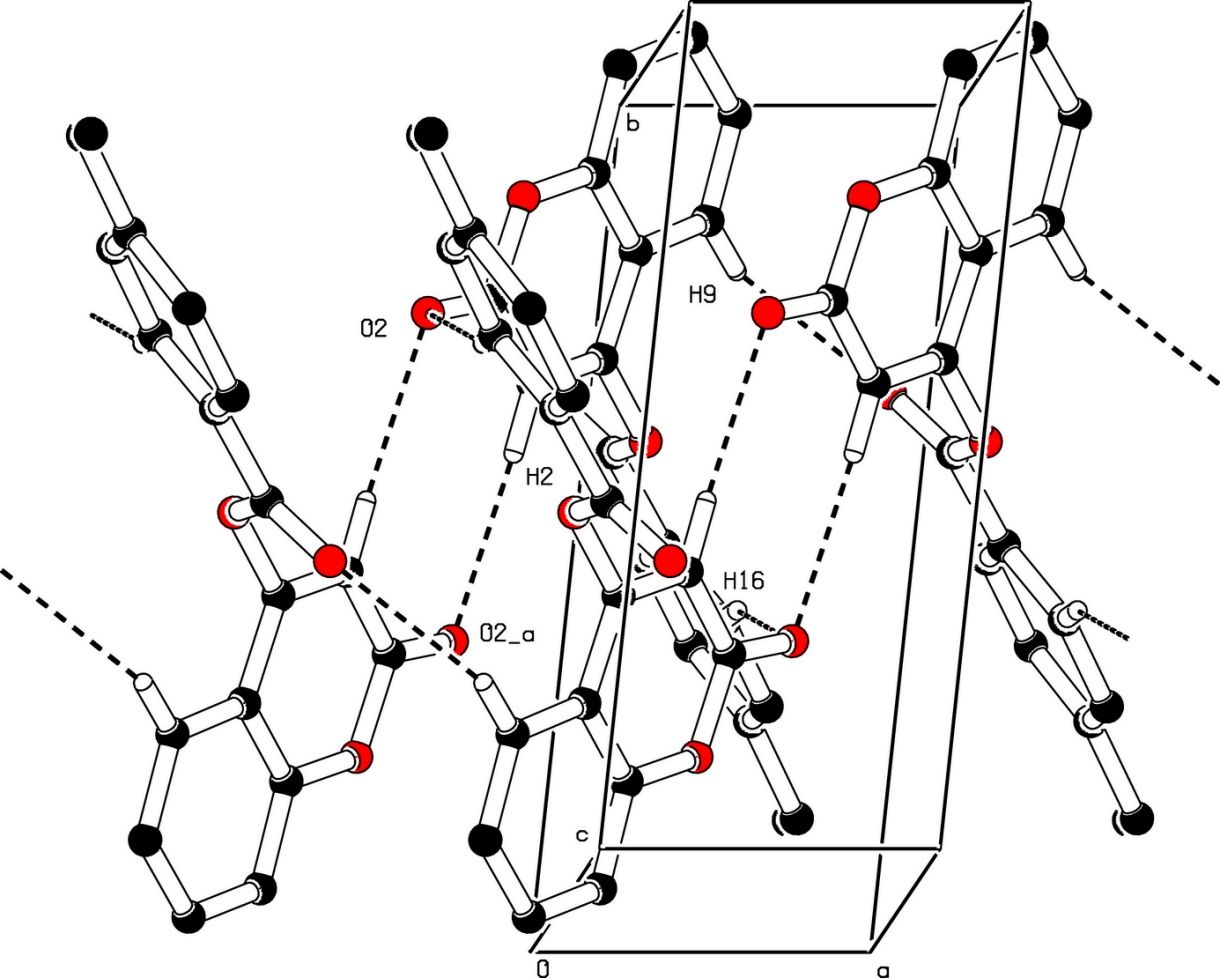
Detail of the packing of (**I**) showing the association of mol­ecules into centrosymmetric dimers through pairwise C—H⋯O hydrogen bonds. This generates 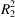
(8) and 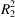
(16) graph-set motifs that extend along the *a*-axis direction. H atoms not involved in hydrogen bonding have been omitted for clarity.

**Table 1:** Hydrogen-bond geometry (Å, °) *Cg*1 is the centroid of the C1–C5/O1 ring.

*D*—H⋯*A*	*D*—H	H⋯*A*	*D*⋯*A*	*D*—H⋯*A*
C2—H2⋯O2^i^	0.93	2.49	3.4223 (14)	176
C9—H9⋯O4^ii^	0.93	2.57	3.4164 (14)	151
C16—H16⋯O2^iii^	0.93	2.54	3.4436 (14)	163
C1—O2⋯*Cg*1^iv^	1.22 (1)	3.27 (1)	3.5408 (14)	93 (1)

Symmetry codes: (i) 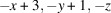; (ii) 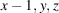; (iii) 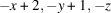; (iv) 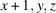.

**Table 2:** Experimental details

Crystal data	
Chemical formula	C_18_H_14_O_4_
*M*_r_	294.29
Crystal system, space group	Triclinic, *P* 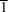
Temperature (K)	296
*a*, *b*, *c* (Å)	4.2781 (4), 10.7096 (9), 15.3525 (13)
α, β, γ (°)	84.816 (3), 86.728 (3), 83.925 (3)
*V* (Å^3^)	695.79 (11)
*Z*	2
Radiation type	Mo *K*α
μ (mm^−1^)	0.10
Crystal size (mm)	0.33 × 0.16 × 0.07

Data collection	
Diffractometer	SuperNova, Dual, Cu at home/near, AtlasS2
Absorption correction	Multi-scan (*CrysAlis PRO*; Rigaku OD, 2022 ▸)
*T*_min_, *T*_max_	0.956, 1.000
No. of measured, independent and observed [*I* > 2σ(*I*)] reflections	59779, 4332, 3675
*R*_int_	0.037
(sin θ/λ)_max_ (Å^−1^)	0.721

Refinement	
*R*[*F*^2^ > 2σ(*F*^2^)], *wR*(*F*^2^), *S*	0.049, 0.144, 1.11
No. of reflections	4332
No. of parameters	200
H-atom treatment	H-atom parameters constrained
Δρ_max_, Δρ_min_ (e Å^−3^)	0.32, −0.28

Computer programs: *CrysAlis PRO* (Rigaku OD, 2022 ▸), *SHELXT2018/2* (Sheldrick, 2015*a* ▸), *PLATON* (Spek, 2020 ▸) and *WinGX* (Farrugia, 2012 ▸), *SHELXL2018/3* (Sheldrick, 2015*b* ▸) and *publCIF* (Westrip, 2010 ▸).

## full crystallographic data

### 2-Oxo-2H-chromen-4-yl 4-ethylbenzoate Crystal data

**Table:**

C_18_H_14_O_4_	*F*(000) = 308
*M**_r_* = 294.29	*D*_x_ = 1.405 Mg m^−^^3^
Triclinic, *P*1	Melting point = 459–461 K
*a* = 4.2781 (4) Å	Mo *K*α radiation, λ = 0.71073 Å
*b* = 10.7096 (9) Å	Cell parameters from 4332 reflections
*c* = 15.3525 (13) Å	θ = 4.5–61.7°
α = 84.816 (3)°	µ = 0.10 mm^−^^1^
β = 86.728 (3)°	*T* = 296 K
γ = 83.925 (3)°	Prism, colorless
*V* = 695.79 (11) Å^3^	0.33 × 0.16 × 0.07 mm
*Z* = 2	

### 2-Oxo-2H-chromen-4-yl 4-ethylbenzoate Data collection

**Table:**

SuperNova, Dual, Cu at home/near, AtlasS2 diffractometer	4332 independent reflections
Radiation source: micro-focus sealed X-ray tube	3675 reflections with *I* > 2σ(*I*)
Detector resolution: 5.3048 pixels mm^-1^	*R*_int_ = 0.037
ω scans	θ_max_ = 30.8°, θ_min_ = 2.2°
Absorption correction: multi-scan (CrysAlisPro; Rigaku OD, 2022)	*h* = −6→6
*T*_min_ = 0.956, *T*_max_ = 1.000	*k* = −15→15
59779 measured reflections	*l* = −22→22

### 2-Oxo-2H-chromen-4-yl 4-ethylbenzoate Refinement

**Table:**

Refinement on *F*^2^	Hydrogen site location: inferred from neighbouring sites
Least-squares matrix: full	H-atom parameters constrained
*R*[*F*^2^ > 2σ(*F*^2^)] = 0.049	*w* = 1/[σ^2^(*F*_o_^2^) + (0.0753*P*)^2^ + 0.2292*P*] where *P* = (*F*_o_^2^ + 2*F*_c_^2^)/3
*wR*(*F*^2^) = 0.144	(Δ/σ)_max_ < 0.001
*S* = 1.11	Δρ_max_ = 0.32 e Å^−^^3^
4332 reflections	Δρ_min_ = −0.28 e Å^−^^3^
200 parameters	Extinction correction: *SHELXL2018/3* (Sheldrick, 2015b), Fc^*^=kFc[1+0.001xFc^2^λ^3^/sin(2θ)]^-1/4^
0 restraints	Extinction coefficient: 0.016 (6)
Primary atom site location: structure-invariant direct methods	

### 2-Oxo-2H-chromen-4-yl 4-ethylbenzoate Special details

**Table:**

Geometry. All esds (except the esd in the dihedral angle between two l.s. planes) are estimated using the full covariance matrix. The cell esds are taken into account individually in the estimation of esds in distances, angles and torsion angles; correlations between esds in cell parameters are only used when they are defined by crystal symmetry. An approximate (isotropic) treatment of cell esds is used for estimating esds involving l.s. planes.
Refinement. The H atoms were placed at calculated positions [C—H = 0.93–0.97 Å] and refined using the riding model approximation with *U*_iso_(H) = 1.2*U*_eq_(C) or 1.5*U*_eq_(methyl C).

### 2-Oxo-2H-chromen-4-yl 4-ethylbenzoate Fractional atomic coordinates and isotropic or equivalent isotropic displacement parameters (Å^2^)

**Table:**

	*x*	*y*	*z*	*U*_iso_*/*U*_eq_	
O1	1.33648 (19)	0.16608 (7)	0.02913 (5)	0.02074 (17)	
O3	0.87511 (19)	0.43581 (7)	0.18225 (5)	0.02160 (18)	
O2	1.5944 (2)	0.31182 (8)	−0.04602 (5)	0.0267 (2)	
O4	1.1515 (2)	0.36404 (8)	0.30123 (5)	0.02480 (19)	
C11	0.7673 (2)	0.54303 (9)	0.30997 (7)	0.0195 (2)	
C3	1.0391 (3)	0.34377 (10)	0.13411 (7)	0.0193 (2)	
C5	1.1274 (2)	0.12891 (10)	0.09534 (7)	0.0187 (2)	
C10	0.9530 (2)	0.43848 (10)	0.26765 (7)	0.0194 (2)	
C2	1.2424 (3)	0.38058 (10)	0.06923 (7)	0.0215 (2)	
H2	1.280225	0.464811	0.060129	0.026*	
C14	0.4429 (3)	0.73961 (10)	0.39781 (7)	0.0207 (2)	
C4	0.9691 (2)	0.21466 (9)	0.15046 (7)	0.0190 (2)	
C7	0.8695 (3)	−0.03992 (10)	0.16993 (7)	0.0232 (2)	
H7	0.834208	−0.124535	0.176664	0.028*	
C13	0.6335 (3)	0.64501 (11)	0.44302 (7)	0.0242 (2)	
H13	0.652704	0.646856	0.502909	0.029*	
C6	1.0813 (3)	0.00155 (10)	0.10473 (7)	0.0209 (2)	
H6	1.190075	−0.054437	0.068072	0.025*	
C17	0.2679 (3)	0.84887 (10)	0.44238 (8)	0.0251 (2)	
H17A	0.050253	0.857056	0.426309	0.030*	
H17B	0.356950	0.925553	0.419603	0.030*	
C15	0.4150 (3)	0.73322 (10)	0.30800 (7)	0.0230 (2)	
H15	0.287568	0.795705	0.277112	0.028*	
C16	0.5729 (3)	0.63586 (10)	0.26384 (7)	0.0214 (2)	
H16	0.549281	0.632588	0.204266	0.026*	
C1	1.4032 (3)	0.28936 (10)	0.01336 (7)	0.0211 (2)	
C12	0.7957 (3)	0.54759 (10)	0.39939 (7)	0.0243 (2)	
H12	0.923771	0.485241	0.430213	0.029*	
C9	0.7567 (3)	0.17035 (10)	0.21634 (7)	0.0214 (2)	
H9	0.648923	0.225798	0.253545	0.026*	
C8	0.7082 (3)	0.04399 (11)	0.22575 (7)	0.0239 (2)	
H8	0.567671	0.014693	0.269405	0.029*	
C18	0.2734 (3)	0.83910 (12)	0.54170 (8)	0.0312 (3)	
H18A	0.156830	0.912340	0.563515	0.047*	
H18B	0.179577	0.765055	0.565512	0.047*	
H18C	0.487067	0.833730	0.558788	0.047*	

### 2-Oxo-2H-chromen-4-yl 4-ethylbenzoate Atomic displacement parameters (Å^2^)

**Table:**

	*U*^11^	*U*^22^	*U*^33^	*U*^12^	*U*^13^	*U*^23^
O1	0.0244 (4)	0.0187 (3)	0.0191 (4)	−0.0020 (3)	0.0021 (3)	−0.0037 (3)
O3	0.0271 (4)	0.0181 (3)	0.0193 (4)	0.0023 (3)	−0.0017 (3)	−0.0051 (3)
O2	0.0312 (4)	0.0266 (4)	0.0220 (4)	−0.0046 (3)	0.0046 (3)	−0.0019 (3)
O4	0.0271 (4)	0.0226 (4)	0.0243 (4)	0.0039 (3)	−0.0043 (3)	−0.0053 (3)
C11	0.0217 (5)	0.0159 (4)	0.0211 (5)	−0.0017 (3)	−0.0009 (4)	−0.0030 (3)
C3	0.0222 (5)	0.0175 (4)	0.0180 (4)	0.0011 (3)	−0.0030 (3)	−0.0038 (3)
C5	0.0196 (4)	0.0188 (4)	0.0177 (4)	−0.0009 (3)	−0.0013 (3)	−0.0023 (3)
C10	0.0218 (5)	0.0176 (4)	0.0191 (4)	−0.0025 (3)	−0.0008 (3)	−0.0029 (3)
C2	0.0260 (5)	0.0180 (4)	0.0207 (5)	−0.0026 (4)	−0.0020 (4)	−0.0023 (3)
C14	0.0228 (5)	0.0164 (4)	0.0231 (5)	−0.0024 (4)	0.0008 (4)	−0.0028 (3)
C4	0.0205 (5)	0.0179 (4)	0.0185 (4)	−0.0008 (3)	−0.0022 (3)	−0.0025 (3)
C7	0.0262 (5)	0.0187 (4)	0.0248 (5)	−0.0034 (4)	−0.0041 (4)	0.0002 (4)
C13	0.0289 (5)	0.0221 (5)	0.0215 (5)	0.0023 (4)	−0.0032 (4)	−0.0057 (4)
C6	0.0232 (5)	0.0173 (4)	0.0223 (5)	−0.0001 (4)	−0.0033 (4)	−0.0036 (3)
C17	0.0292 (6)	0.0182 (5)	0.0271 (5)	0.0011 (4)	0.0019 (4)	−0.0042 (4)
C15	0.0277 (5)	0.0169 (4)	0.0235 (5)	0.0010 (4)	−0.0018 (4)	−0.0009 (4)
C16	0.0265 (5)	0.0183 (4)	0.0193 (4)	−0.0013 (4)	−0.0012 (4)	−0.0015 (3)
C1	0.0239 (5)	0.0200 (5)	0.0192 (4)	−0.0022 (4)	−0.0016 (4)	−0.0012 (3)
C12	0.0289 (5)	0.0205 (5)	0.0230 (5)	0.0037 (4)	−0.0046 (4)	−0.0041 (4)
C9	0.0220 (5)	0.0222 (5)	0.0200 (5)	−0.0018 (4)	−0.0006 (4)	−0.0027 (4)
C8	0.0249 (5)	0.0249 (5)	0.0221 (5)	−0.0057 (4)	−0.0012 (4)	0.0004 (4)
C18	0.0400 (7)	0.0250 (5)	0.0277 (6)	0.0048 (5)	0.0022 (5)	−0.0085 (4)

### 2-Oxo-2H-chromen-4-yl 4-ethylbenzoate Geometric parameters (Å, º)

**Table:**

O1—C5	1.3750 (13)	C7—C6	1.3861 (15)
O1—C1	1.3775 (13)	C7—C8	1.3995 (16)
O3—C10	1.3747 (13)	C7—H7	0.9300
O3—C3	1.3915 (12)	C13—C12	1.3924 (15)
O2—C1	1.2146 (13)	C13—H13	0.9300
O4—C10	1.2062 (13)	C6—H6	0.9300
C11—C12	1.3908 (15)	C17—C18	1.5202 (17)
C11—C16	1.3954 (15)	C17—H17A	0.9700
C11—C10	1.4802 (14)	C17—H17B	0.9700
C3—C2	1.3443 (15)	C15—C16	1.3891 (15)
C3—C4	1.4425 (14)	C15—H15	0.9300
C5—C6	1.3922 (14)	C16—H16	0.9300
C5—C4	1.4004 (14)	C12—H12	0.9300
C2—C1	1.4508 (15)	C9—C8	1.3842 (15)
C2—H2	0.9300	C9—H9	0.9300
C14—C13	1.3912 (15)	C8—H8	0.9300
C14—C15	1.3991 (15)	C18—H18A	0.9600
C14—C17	1.5161 (15)	C18—H18B	0.9600
C4—C9	1.4047 (15)	C18—H18C	0.9600
			
C5—O1—C1	122.05 (8)	C5—C6—H6	120.7
C10—O3—C3	117.26 (8)	C14—C17—C18	115.86 (9)
C12—C11—C16	119.86 (10)	C14—C17—H17A	108.3
C12—C11—C10	117.31 (9)	C18—C17—H17A	108.3
C16—C11—C10	122.83 (9)	C14—C17—H17B	108.3
C2—C3—O3	118.14 (9)	C18—C17—H17B	108.3
C2—C3—C4	122.17 (9)	H17A—C17—H17B	107.4
O3—C3—C4	119.51 (9)	C16—C15—C14	121.64 (10)
O1—C5—C6	116.49 (9)	C16—C15—H15	119.2
O1—C5—C4	121.75 (9)	C14—C15—H15	119.2
C6—C5—C4	121.75 (10)	C15—C16—C11	119.17 (10)
O4—C10—O3	122.50 (9)	C15—C16—H16	120.4
O4—C10—C11	126.07 (10)	C11—C16—H16	120.4
O3—C10—C11	111.42 (9)	O2—C1—O1	116.76 (10)
C3—C2—C1	120.23 (10)	O2—C1—C2	125.71 (10)
C3—C2—H2	119.9	O1—C1—C2	117.53 (9)
C1—C2—H2	119.9	C11—C12—C13	120.35 (10)
C13—C14—C15	118.37 (10)	C11—C12—H12	119.8
C13—C14—C17	122.29 (10)	C13—C12—H12	119.8
C15—C14—C17	119.34 (10)	C8—C9—C4	119.86 (10)
C5—C4—C9	118.73 (9)	C8—C9—H9	120.1
C5—C4—C3	116.26 (9)	C4—C9—H9	120.1
C9—C4—C3	125.01 (9)	C9—C8—C7	120.35 (10)
C6—C7—C8	120.80 (10)	C9—C8—H8	119.8
C6—C7—H7	119.6	C7—C8—H8	119.8
C8—C7—H7	119.6	C17—C18—H18A	109.5
C14—C13—C12	120.59 (10)	C17—C18—H18B	109.5
C14—C13—H13	119.7	H18A—C18—H18B	109.5
C12—C13—H13	119.7	C17—C18—H18C	109.5
C7—C6—C5	118.50 (10)	H18A—C18—H18C	109.5
C7—C6—H6	120.7	H18B—C18—H18C	109.5
			
C10—O3—C3—C2	−110.14 (11)	C8—C7—C6—C5	−0.71 (16)
C10—O3—C3—C4	74.63 (12)	O1—C5—C6—C7	−179.21 (9)
C1—O1—C5—C6	−179.77 (9)	C4—C5—C6—C7	0.81 (16)
C1—O1—C5—C4	0.22 (15)	C13—C14—C17—C18	−8.76 (16)
C3—O3—C10—O4	−0.98 (15)	C15—C14—C17—C18	171.64 (11)
C3—O3—C10—C11	178.33 (8)	C13—C14—C15—C16	−0.28 (17)
C12—C11—C10—O4	−8.15 (17)	C17—C14—C15—C16	179.33 (10)
C16—C11—C10—O4	170.94 (11)	C14—C15—C16—C11	−0.84 (17)
C12—C11—C10—O3	172.57 (9)	C12—C11—C16—C15	1.29 (17)
C16—C11—C10—O3	−8.34 (14)	C10—C11—C16—C15	−177.78 (10)
O3—C3—C2—C1	−175.83 (9)	C5—O1—C1—O2	179.08 (9)
C4—C3—C2—C1	−0.73 (16)	C5—O1—C1—C2	−0.34 (15)
O1—C5—C4—C9	179.49 (9)	C3—C2—C1—O2	−178.76 (11)
C6—C5—C4—C9	−0.53 (16)	C3—C2—C1—O1	0.60 (16)
O1—C5—C4—C3	−0.30 (15)	C16—C11—C12—C13	−0.62 (17)
C6—C5—C4—C3	179.68 (9)	C10—C11—C12—C13	178.49 (10)
C2—C3—C4—C5	0.57 (15)	C14—C13—C12—C11	−0.52 (18)
O3—C3—C4—C5	175.61 (9)	C5—C4—C9—C8	0.14 (16)
C2—C3—C4—C9	−179.20 (10)	C3—C4—C9—C8	179.91 (10)
O3—C3—C4—C9	−4.17 (16)	C4—C9—C8—C7	−0.05 (16)
C15—C14—C13—C12	0.96 (17)	C6—C7—C8—C9	0.34 (17)
C17—C14—C13—C12	−178.64 (11)		

### 2-Oxo-2H-chromen-4-yl 4-ethylbenzoate Hydrogen-bond geometry (Å, º)

*Cg*1 is the centroid of the C1–C5/O1 ring.

**Table:**

*D*—H···*A*	*D*—H	H···*A*	*D*···*A*	*D*—H···*A*
C2—H2···O2^i^	0.93	2.49	3.4223 (14)	176
C9—H9···O4^ii^	0.93	2.57	3.4164 (14)	151
C16—H16···O2^iii^	0.93	2.54	3.4436 (14)	163
C1—O2···*Cg*1^iv^	1.22 (1)	3.27 (1)	3.5408 (14)	93 (1)

Symmetry codes: (i) −*x*+3, −*y*+1, −*z*; (ii) *x*−1, *y*, *z*; (iii) −*x*+2, −*y*+1, −*z*; (iv) *x*+1, *y*, *z*.

## References

[bb1] Akkol, E. K., Genç, Y., Karpuz, B., Sobarzo-Sánchez, E. & Capasso, R. (2020). *Cancers (Basel)* **12**, 1–25. 10.3390/cancers12071959 PMC7409047 32707666

[bb2] Farrugia, L. J. (2012). *J. Appl. Cryst.* **45**, 849–854.

[bb3] Gomes, L. R., Low, J. N., Fonseca, A., Matos, M. J. & Borges, F. (2016). *Acta Cryst.* E**72**, 926–932. 10.1107/S2056989016008665 PMC4992908 27555933

[bb4] Koulabiga, Z., Yao, K. H., Abou, A., Djandé, A., Giorgi, M. & Coussan, S. (2024). *Am. J. Org. Chem.* **12**, 1–19.

[bb5] Rigaku OD (2022). *CrysAlis PRO.* Rigaku Oxford Diffraction, Yarnton, England.

[bb6] Sheldrick, G. M. (2015*a*). *Acta Cryst.* A**71**, 3–8.

[bb7] Sheldrick, G. M. (2015*b*). *Acta Cryst.* C**71**, 3–8.

[bb8] Spek, A. L. (2020). *Acta Cryst.* E**76**, 1–11. 10.1107/S2056989019016244 PMC6944088 31921444

[bb9] Tosun, A., Akkol, E. K. & Yesilada, E. (2009). *Z. Naturforsch., C: J. Biosci.* **64**, 56–62. 10.1515/znc-2009-1-210 19323267

[bb10] Tuan Anh, H. L., Kim, D.-C., Ko, W., Ha, T. M., Nhiem, N. X., Yen, P. H., Tai, B. H., Truong, L. H., Long, V. N., Gioi, T., Hong Quang, T., Minh, C. V., Oh, H., Kim, Y. C. & Kiem, P. V. (2017). *Pharm. Biol.* **55**, 1195–1201. 10.1080/13880209.2017.1296001 PMC6130569 28245363

[bb11] Westrip, S. P. (2010). *J. Appl. Cryst.* **43**, 920–925.

[bb12] Ziarani, G. M., Moradi, R., Lashgari, N. & Kruger, H. G. (2018). *Metal-Free Synthetic Organic Dyes* ch. 7, *Coumarin dyes* pp. 117–125. https://doi.org/10.1016/b978-0-12-815647-6.00007-8.

